# HER2 double-equivocal breast cancer in Chinese patients: a high concordance of HER2 status between different blocks from the same tumor

**DOI:** 10.1007/s10549-019-05387-6

**Published:** 2019-08-06

**Authors:** Yuanyuan Liu, Shafei Wu, Xiaohua Shi, Yufeng Luo, Junyi Pang, Changjun Wang, Feng Mao, Zhiyong Liang, Xuan Zeng

**Affiliations:** 1 Molecular Pathology Research Center, Department of Pathology, Peking Union Medical College Hospital, Peking Union Medical College, Chinese Academy of Medical Sciences, Beijing, China; 2 Department of Breast Surgery, Peking Union Medical College Hospital, Peking Union Medical College, Chinese Academy of Medical Sciences, Beijing, China

**Keywords:** Breast cancer, HER2, Equivocal, IHC, FISH

## Abstract

**Purpose:**

Human epidermal growth factor receptor 2 (HER2) status is both an independent prognostic factor and a predictive factor for the efficacy of targeted therapy for breast cancer, so it is critical to accurately detect HER2 protein expression and/or gene amplification. According to the recommendations of the 2013 American Society of Clinical Oncology and College of American Pathologists (ASCO/CAP) guidelines for HER2 breast cancer testing, an additional test should be pursued on a different block from the same tumor as one of the options for patients with immunohistochemistry (IHC) 2+ and a HER2/CEP17 ratio of < 2.0 with an average HER2 signals per tumor cell of ≥ 4.0 and < 6.0 by reflex test using dual-probe fluorescence in situ hybridization (FISH) (double-equivocal HER2). Our aim in this study is to explore the consistency of HER2 status between the two blocks.

**Methods:**

We retrospectively analyzed 5685 primary invasive breast cancers between April 2015 and January 2019 from Peking Union Medical College Hospital. For cases with double-equivocal HER2 revealed in initial blocks, HER2 gene status was evaluated by FISH in a different block from the same tumor. The FISH score was interpreted according to the 2013 ASCO/CAP guidelines for HER2 testing.

**Results:**

In our cohort of 5685 specimens, the overall HER2 IHC3+, 2+, 1+ and 0 cases were 20.5%, 31.8%, 28.3%, and 19.5%, respectively. Then, 13.7%, 66.3%, and 20.0% of HER2 amplification, non-amplification, and equivocation rates were found, respectively, in IHC2+ patients (*n* = 1777) by reflex FISH assay. For specimens with double-equivocal HER2 (*n* = 333), HER2 status was assessed in another block from the same tumor by FISH and then the frequency of HER2 positive, negative, and equivocation was estimated at 5.7%, 22.5%, and 71.8%, respectively. Because double-equivocal HER2 cases are classified in the HER2 negative category by the 2018 ASCO/CAP HER2 testing guidelines, only 1.3% (19/1511) of HER2 positive patients were determined through additional HER2 testing in another block from the HER2 negative population.

**Conclusions:**

HER2 status in different blocks from the same tumor in primary invasive breast cancer was highly concordant. Our data supported the recommendation of the 2018 ASCO/CAP HER2 testing guidelines in breast cancer to remove the suggestion for additional HER2 testing using another block contained in the previous version.

## Introduction

Human epidermal growth factor receptor 2 (HER2) is a transmembrane protein that plays a key role in the regulation of cell growth, apoptosis, and differentiation, so overactivity of HER2 will lead to malignant biological behavior in the breast [1]. HER2 gene amplification and/or protein overexpression occurs in approximately 25–30% of invasive breast cancer [2, 3]. Studies show that HER2 is not only an independent factor of poor prognosis, but also a crucial predictive factor for the response to treatment, especially for anti-HER2 targeted therapy, e.g., trastuzumab, pertuzumab, lapatinib, and Trastuzumab–emtansine, which have been demonstrated to produce responses in breast cancers showing an HER2-positive feature [4]. Therefore, it is necessary to accurately determine HER2 status for each breast cancer patient.

Currently, there are two common methods of HER2 detection. One is immunohistochemistry (IHC) for HER2 protein expression examination, and another is fluorescent in situ hybridization (FISH) for HER2 gene amplification examination. In most laboratories in China, IHC analysis is generally used as a primary screening for HER2 status, and the reflex test would be performed by FISH for HER2 IHC2+ cases, although both assays were implemented simultaneously in a small number of laboratories.

HER2 status should be reported as an equivocal result if an HER2/CEP17 ratio < 2.0 with an average HER2 copy number ≥ 4.0 and < 6.0 per tumor cell is detected by dual-probe FISH assay, according to the 2013 ASCO/CAP HER2 testing guidelines. In order to obtain a definitive HER2 result, an additional test was recommended according to these guidelines. They recommended using the FISH or IHC method and another formalin-fixed paraffin-embedded (FFPE) block from the same tumor, or a new sample (if available) for double-equivocal (both IHC and FISH equivocal) cases because the decision to use HER2-targeted therapy for eligible patients requires an exact HER2-positive result. However, the recommendation of an additional test for HER2 double-equivocal cases in the 2013 version was abolished in the updated 2018 ASCO/CAP HER2 testing guidelines (HER2 was classified as 5 groups: group 1 means HER2/CEP17 ratio ≥ 2.0 with an average HER2 signals/cell ratio ≥ 4.0; group 2 means HER2/CEP17 ratio ≥ 2.0 with an average HER2 signals/cell < 4.0; group 3 means HER2/CEP17 ratio < 2.0 with an average HER2 signals/cell ≥ 6.0; group 4 means HER2/CEP17 ratio < 2.0 with an average HER2 signals/cell ≥ 4.0 and < 6.0; group 5 means HER2/CEP17 ratio < 2.0 with an average HER2 signals/cell < 4.0). However, sufficient supporting data were not shown [5, 6] for either the additional HER2 test recommended in the previous guideline or the opposite recommendation in the update version.

Studies have shown that the HER2 IHC2+ rate is about 17% in breast cancer [7], but a small number of ambiguous cases for HER2 status still remained after reflex FISH testing. This unresolved question brought confusion and clinical challenges to pathologists, oncologists, and patients about prognosis evaluation and HER2-targeted therapies, in spite of the relatively low incidence of HER2 double-equivocal subtype compared with other molecular groups in breast cancer. As FISH assay is accepted as a gold standard for HER2 status determination, we detected HER2 gene status by FISH using another FFPE block from the same tumor, which was available and examined successfully for the most of cases in daily clinical practice.

We analyzed 333 cases of HER2 status in another FFPE block from the same tumor with double-equivocal HER2 in initial specimens identified from 5685 invasive primary breast cancers according to the 2013 ASCO/CAP guidelines. The purpose of our study is to evaluate the necessity of HER2 assessment using another block from the same tumor for HER2 double-equivocal cases in the first FFPE blocks.

## Materials and methods

### Patient population

A total of 5685 consecutive primary invasive breast cancers archived in Peking Union Medical College Hospital between April 2015 and January 2019 were retrospectively analyzed. They included 5038 invasive breast carcinoma of no specific type (IBC-NST), 190 invasive lobular carcinoma (ILC), 9 tubular carcinoma, 114 mucinous carcinoma (MC), 18 invasive cribriform carcinoma (ICC), 163 mixed ductal/lobular carcinoma, 62 invasive micropapillary carcinoma (IMPC), 32 carcinoma with medullary features (CMF), and 59 carcinoma of breast with neuroendocrine differentiation. First, IHC was carried out on FFPE samples (including core biopsies and surgical resection) in an initial survey to reveal HER2 expression, and then reflex FISH assay was performed to explore HER2 amplification for IHC-equivocal (2+) cases. Moreover, FISH was performed again for the cases in which HER2 status was categorized as FISH equivocal by the 2013 guidelines (FISH group 4 by 2018 guidelines) using another FFPE block from the same tumor.

### Immunohistochemistry for HER2 expression

HER2 protein expression was inspected on FFPE sections at 4 μm thickness with Ventana Ultra autostainer platform (Ventana Medical Systems, Inc., Tucson, AZ, USA) using the antibody of clone 4B5 according to the standard autostaining procedure. IHC slides was scored as 0 (No staining, or membrane staining that is incomplete and is faint/barely perceptible and within ≤ 10%); 1+ (Incomplete membrane staining that is faint/barely perceptible and within > 10% of tumor cells); 2+ (Circumferential membrane staining that is incomplete and/or weak/moderate and within > 10% of tumor cells or complete and circumferential membrane staining that is intense and within ≤ 10% of tumor cells); or 3+ (circumferential membrane staining that is complete, intense, and within > 10% of tumor cells) according to the 2013 ASCO/CAP guidelines [5]. IHC3+ means HER2 positive, IHC1+ and IHC0 was defined as HER2 negative, and IHC2+ was categorized as HER2-equivocal and needed to be reflex tested by FISH.

### Fluorescence in situ hybridization for HER2 amplification

The FISH test was performed on FFPE slides with a thickness of 4–5 μm using PathVysion HER2 DNA probe kit (Vysis/Abbott, Abbott Park, Illinois) based on the ThermoBrite Elite automated FISH slides prep system (Leica, Richmond, CA, USA) according to the instruction manual. HER2 and CEP 17 signals were counted in at least 20 cell nuclei from at least two areas of invasive tumor under the CytoVision DM6000B fluorescent microscope system (Leica, Biosystem, Buffalo Grove, IL). The interpretation criteria of FISH signals for positive, negative, and equivocation were recognized as follows: a ratio of HER2/CEP17 ≥ 2.0 or an average of HER2 signals/cell ≥ 6.0 with a HER2/CEP17 ratio of < 2.0; a ratio of HER2/CEP17 < 2.0 with an average of HER2 signals/cell < 4.0; and an average of HER2 signals/cell ≥ 4.0 and < 6.0 HER2 with a HER2/CEP17 ratio of < 2.0, respectively, according to the 2013 ASCO/CAP HER2 test guidelines [5].

## Results

**Fig. 1:**
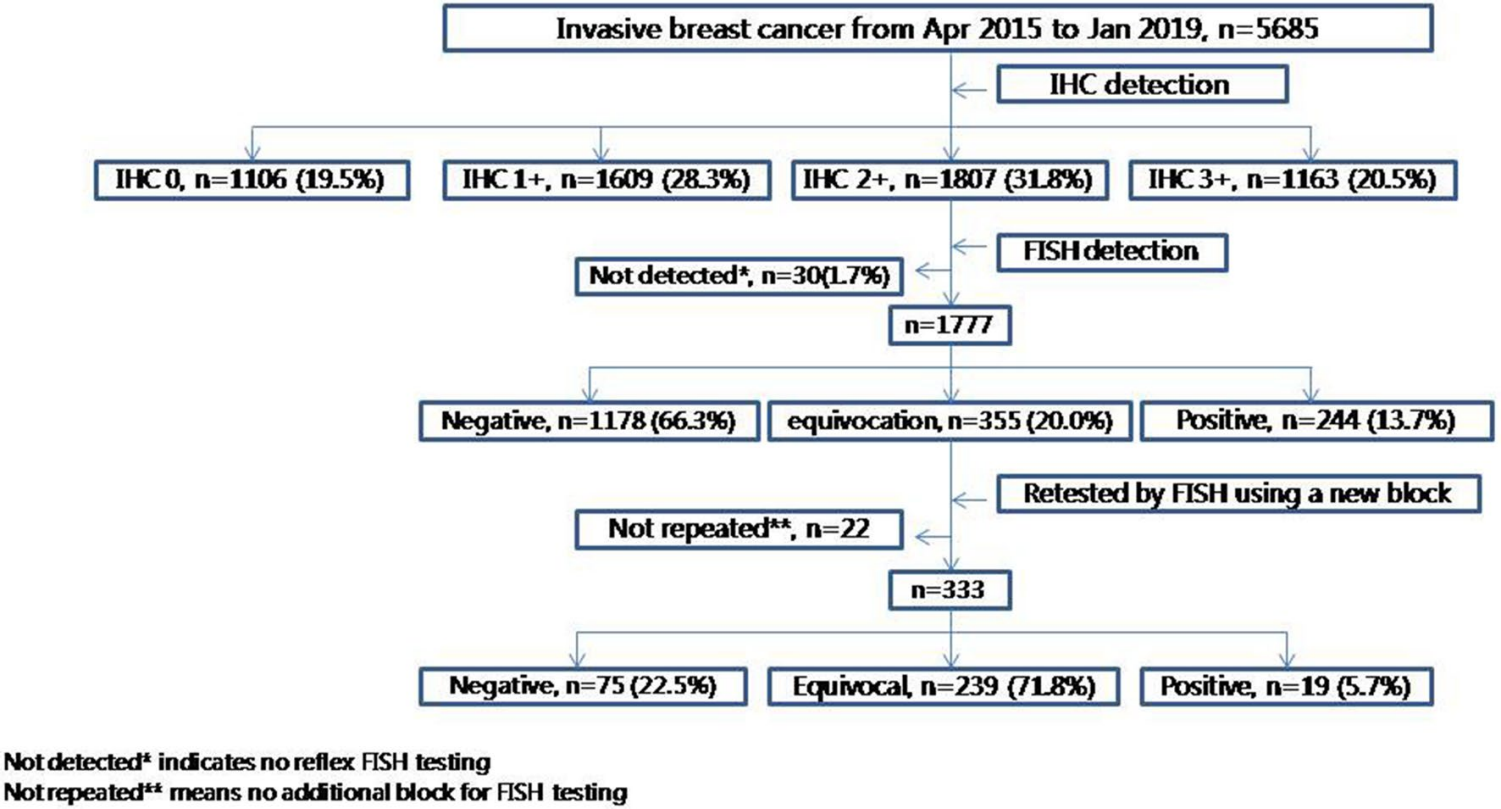
Study algorithm

In all 5685 breast cancer cases, 1163 of IHC3+ (20.5%), 1807 of IHC2+ (31.8%), 1609 of IHC1+ (28.3%), and 1106 of IHC0 (19.5%) were found. In total, 1777 IHC2+ cases were analyzed by reflex FISH test (30 HER2 IHC-equivocal cases failed to be re-tested for various reasons) for determining HER2 status. FISH positive, negative and equivocation was uncovered in 244 of 1777 (13.7%), 1178 of 1777 (66.3%), and 355 of 1777 (20.0%), respectively. Of 333 HER2 double-equivocal cases analyzed by reflex FISH testing (22 HER2 double-equivocal cases failed to be tested because no additional blocks were available) using another FFPE block from the same tumor, 19 (5.7%, 19/333) positive, 75 (22.5%, 75/333) negative, and 239 (71.8%, 239/333) equivocation of HER2 status were found (Fig. 1).

**Fig. 2:**
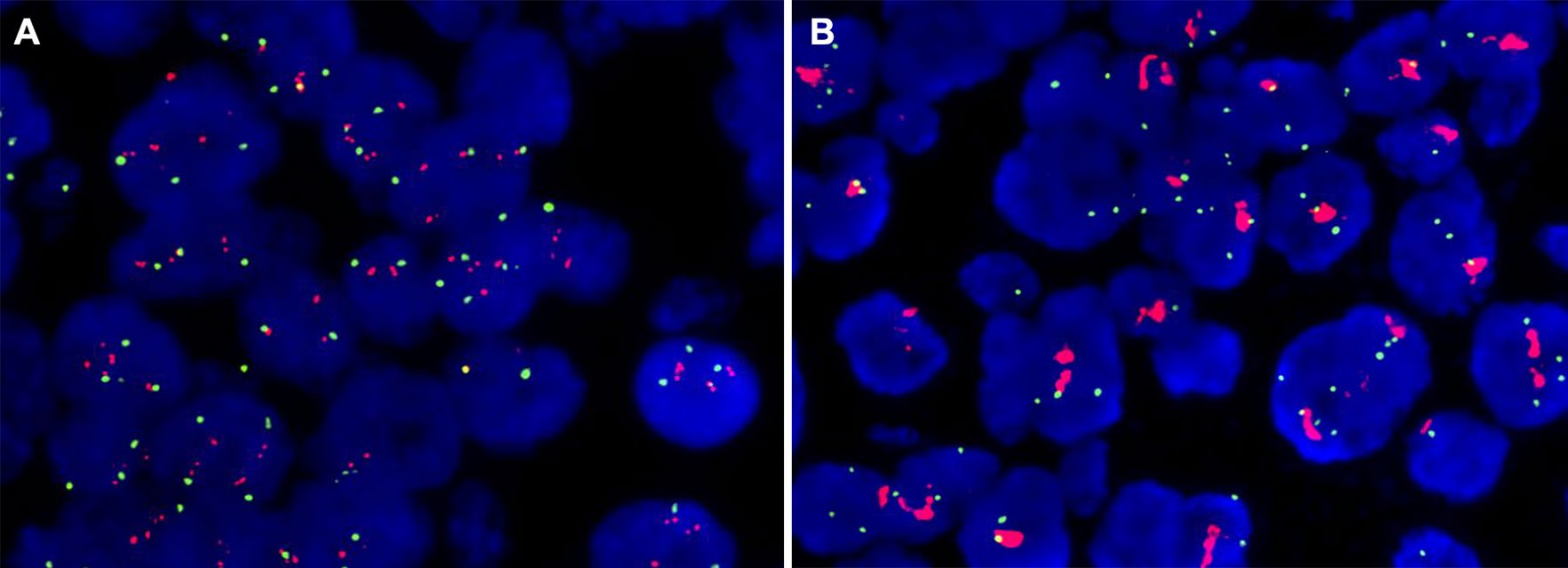
Representative FISH images from case 6: HER2/CEP17 ratio 1.77 in the first block (**a**) but 5.10 in the second block (**b**)

Through re-testing HER2 status by FISH in another block, 19 out of 333 cases of HER2 were identified positive for HER2 double-equivocal cases in the previous block. In these HER2 positive samples, 8 of 19 had a ratio of HER2/CEP17 ≥ 2.0, with the range from 2.00 to 5.10, while HER2 status had a ratio of HER2/CEP17 between 1.25 and 1.95 in the previous blocks. The case with the lowest HER2/CEP17 ratio of 1.25 in the first block reached 2.11 in the second block. The biggest difference of HER2/CEP17 ratio in the case was of 1.77 in the first block, and 5.10 in the second block by FISH assessment (Fig. 2).

**Fig. 3:**
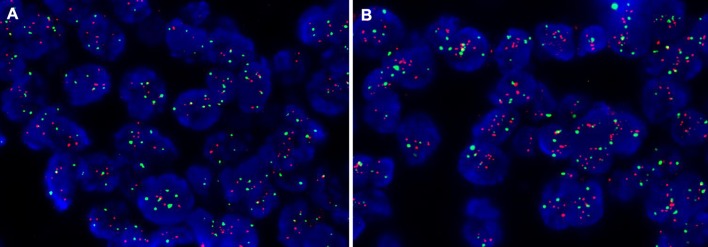
Representative FISH images from case 19: HER2/CEP17 ratio < 2.0 with an average HER2 signal/cell of 5.85 (**a**) in the first block but 9.75 in the second block (**b**)

However, another 11 FISH positive cases all had a HER2/CEP17 ratio < 2.0 with an average of HER2 signals/cell ≥ 6.0, which were between 4.20 and 5.85 in the first blocks and between 6.05 and 9.75 in the second blocks. The biggest difference of the HER2 status in the case was an average HER2 gene copy number per tumor cell of 5.85 in the first block and 9.75 in the second (Fig. 3). HER2 double-equivocal (IHC2+ and FISH group 4) cases were classified as HER2 negative according to the 2018 ASCO/CAP guidelines of HER2 testing in breast cancer, so only 1.3% HER2 positive patients (19) were determined in the HER2 negative population (1533-22) by additional FISH test using another block from the same tumor.

**Fig. 4:**
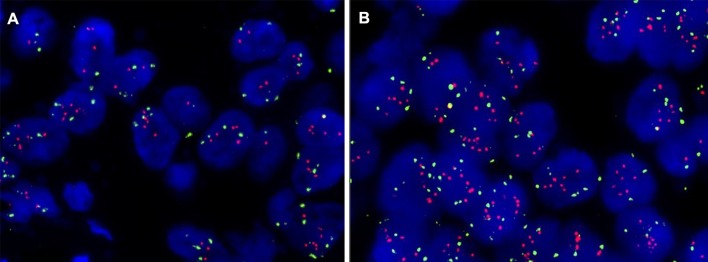
Representative FISH images from a mucinous carcinoma (case 13) with HER2 positive in second block (**b** HER2/CEP17 ratio < 2.0 with an average HER2 signal/cell of 6.05) compared with the result in the first block (**a** HER2/CEP17 ratio < 2.0 with an average HER2 signal/cell of 5.30)

There were 16 IBC-NST, 1 IMPC, 1 MC (Fig. 4), and 1 ILC in a total of 19 HER2 positive cases by HER2 re-testing in another block. HER2 status and histological types of these HER2 positive cases are listed in (Table 1).

**Table 1:** Detailed results of 19 HER2 positive cases using blocks by additional FISH testing

FISH testing using another block	Number of cases	FISH result from first block (average HER2 signal/average CEP17 signal = ratio)	FISH result from second block (average HER2 signal/average CEP17 signal = ratio)	Histopathological types
HER2/CEP17 ratio ≥ 2.0	8	① 5.43/2.98 = 1.82	① 5.38/2.60 = 2.07	IBC-NST
		② 5.38/2.75 = 1.95	② 5.00/2.05 = 2.44	IBC-NST
		③ 4.45/2.40 = 1.85	③ 4.78/2.10 = 2.27	IBC-NST
		④ 5.00/2.95 = 1.69	④ 5.25/2.63 = 2.00	IBC-NST
		⑤ 4.33/2.85 = 1.52	⑤ 7.00/2.95 = 2.37	IMPC
		⑥ 4.70/2.65 = 1.77	⑥ 13.25/2.60 = 5.10	IBC-NST
		⑦ 4.35/3.48 = 1.25	⑦ 6.45/3.05 = 2.11	IBC-NST
		⑧ 4.45/2.40 = 1.85	⑧ 5.40/2.65 = 2.04	IBC-NST
HER2/CEP17 ratio < 2.0 with HER2 signal/cell ≥ 6.0	11	⑨ 4.20/3.28 = 1.28	⑨ 6.45/4.35 = 1.48	IBC-NST
		⑩ 5.55/3.80 = 1.46	⑩ 6.55/5.20 = 1.26	IBC-NST
		⑪ 4.70/2.58 = 1.83	⑪ 6.70/3.40 = 1.97	IBC-NST
		⑫ 5.25/3.20 = 1.64	⑫ 6.05/3.70 = 1.64	IBC-NST
		⑬ 5.30/5.05 = 1.05	⑬ 6.05/5.05 = 1.20	MC
		⑭ 5.45/3.35 = 1.63	⑭ 7.40/4.00 = 1.85	IBC-NST
		⑮ 5.18/3.03 = 1.71	⑮ 6.05/3.50 = 1.73	ILC
		⑯ 5.20/2.93 = 1.78	⑯ 6.15/3.50 = 1.76	IBC-NST
		⑰5.43/4.80 = 1.13	⑰6.75/5.80 = 1.16	IBC-NST
		⑱ 4.53/2.43 = 1.87	⑱7.53/3.90 = 1.93	IBC-NST
		⑲5.85/5.10 = 1.15	⑲ 9.75/5.95 = 1.64	IBC-NST

## Discussion

This is a single center study in which HER2 status was detected using different blocks from the same tumor of primary invasive breast cancer with HER2 double-equivocal in a larger sample size. HER2 protein expression was first measured by IHC for all patients. In our cohort of 5685, HER2 IHC3+, 2+, 1+, and 0 was observed in 20.5%, 31.8%, 28.3%, and 19.5% cases, respectively. Varga Z et al. reported that HER2 status was IHC3+ 21.5%, IHC2+ 46.5%, IHC1+ 22.3%, and IHC 0 3.8% in 1024 cases, respectively, in a retrospective data analysis. The proportion of IHC3+ and IHC1+ cases was relatively close to our study, while there was some difference in the incidence of IHC2+ and IHC0 between the two studies (probably because of the ethnic diversity, different antibody used or a different interpretation criteria for IHC scoring) [8].

In the 1777 specimens with inconclusive HER2 IHC results (2+), 13.7% HER2 amplification, 66.3% non-amplification, and 20.0% equivocation was revealed by reflex FISH testing, respectively. Data from the retrospective multicenter study in China showed that the percentage of HER2 amplification, non-amplification and equivocation in the IHC2+ population were 17.8%, 76.2%, and 6%, respectively. The differences of the frequencies in the subgroups between their study and ours may be ascribed to the data sets being from multicenter versus single center [9].

In 333 HER2 double-equivocal cases, we detected HER2 status in another block from the same tumor by FISH and recognized 5.7%, 22.5%, and 71.8% of cases as HER2 amplification, non-amplification, and equivocation, respectively. The large majority of cases still remained indeterminate HER2 status after duplicate detection using different blocks.

In terms of 19 HER2 positive cases detected in another block, the average HER2 signals/cell (FISH group 3) or HER2/CEP17 ratio (FISH group 1) in the second blocks was similar to the first blocks for the majority of patients (Table 1). This slight difference between separate blocks can probably be attributed to the randomness of the tumor cells counted and was not due to heterogeneity in breast cancer. In addition, 8 of 19 cases exhibited an HER2/CEP17 ratio ≥ 2.0 detected in another block, including 2 cases with obvious differences in the ratio of HER2/CEP17 between two blocks; 11 of 19 cases had an HER2/CEP 17 ratio < 2.0 with an average HER2 signals/cell ≥ 6.0 (FISH group 3), including only 2 cases with a remarkable difference of average HER2 gene copy number/cell between two blocks. For the four HER2 positive cases with distinctly different HER2 results in the two blocks above, there were probable genetic heterogeneity in the primary tumors, in which this kind of molecular feature was reported in previous studies [10, 11].

Although the reason for the discordant HER2 status between two blocks from the same tumor in primary breast cancer was not quite clear, intratumoral heterogeneity may be one of the speculated major causes. The intratumoral heterogeneity of HER2 in breast cancer was observed in previous studies [11]. The exact incidence of the regional difference of HER2 status in primary breast cancer with two different populations of cancer cells (one is HER2-negative and another is HER2-positive) was unknown, but it may be revealed by detecting more than one blocks. Adem et al. assessed heterogeneity in 53 cases of sporadic invasive breast cancers in whole slides prepared from multiple blocks of the same patients; however, no obvious intratumoral heterogeneity was found, including HER2 gene amplification [12]. After another block was detected for the cases with double-equivocal HER2 status in 333 breast cancers in initial blocks of our cohort, 19 HER2 amplification was distinguished (5.7%, 19/333), including 8 FISH group 1 and 11 FISH group 3, and only 4 cases of obvious different results of HER2 status between two blocks was found (1.2%, 4/333) (cases 5, 6, 18, 19 in Table 1). Consequently, cases with different HER2 status in two blocks were something of a rarity. Therefore, the difference of HER2 results between two blocks was quite limited. There was basically little impact on the final results of HER2 status for testing different blocks and interpreting different slides (random counting of at least 20 cancer cells in at least 2 invasive regions, according to the criteria) for a vast majority of cases without intratumoral heterogeneity. This was first single-institution, large sample size analysis of HER2 heterogeneity in different blocks from the same tumors in primary invasive breast cancer.

In addition, one HER2 positive mucinous carcinoma from a 40-year-old woman was determined in another block from 333 double-equivocal HER2 status samples (HER2 positive rate in mucinous carcinoma was 0.70%, 1/144 in our cohort). Although this histopathological type of breast cancer had a good prognosis and was rarely HER2 positive in most cases [13], axillary lymph node metastasis and high ki67 index (50%) was displayed besides the primary tumor with a size of 2 × 1 × 1 cm^3^ in this patient. The HER2/CEP17 ratio was < 2.0 with an average HER2 signal/cell of 5.30 in the first block, and then up to an average HER2 signal/cell of 6.05 was detected in another block (eventually classified as HER2 positive for this woman, despite a slight difference between two blocks). Although the patient had a pathological and molecular phenotype of potentially poor prognosis, it was a matter worthy of attention. Garcia Hernandez I reported a 48-year-old HER2 positive mucinous carcinoma patient who received neoadjuvant chemotherapy based on trastuzumab and pertuzumab, but she had almost no pathological response [14].

According to the 2018 ASCO/CAP HER2 testing guidelines, cases with IHC2+ and FISH Group 4 are classified as HER2-negative, so 13.7% (244/1777) HER2 positive and 86.3% [(1178 + 355)/1777] HER2 negative cases were determined in the total of 1777 IHC2+ patients by reflex FISH test in our study. Furthermore, only 1.3% (19/1511) HER2 positive cases were identified from the total HER2 negative population, with most of the slight difference in HER2 score between two blocks probably resulting from the occasionality in cells counted in some cases.

In the process of re-testing HER2 status with another block by FISH for HER2 double-equivocal cases, costs were high in terms of manpower and time, but the results were not hugely different from the previous test, with a high concordance of HER2 status between two blocks from the same tumors. Therefore, it was not cost-effective to re-examine HER2 double-equivocal specimens with another block. Our data supported the abolishment of the recommendation for additional tests in double-equivocal HER2 status (the suggestion in the 2013 guidelines) in the 2018 ASCO/CAP guidelines. It was not necessary for re-testing another block from the same tumor for double-equivocal HER2 cases in primary breast cancer.

## Abbreviations

HER2: Human epidermal growth factor receptor 2
ASCO: American Society of Clinical Oncology
CAP: College of American Pathologists
IHC: Immunohistochemistry
FISH: Fluorescence in situ hybridization
FFPE: Formalin-fixed paraffin-embedded
IBC-NST: Invasive breast carcinoma of no specific type
ILC: Invasive lobular carcinoma
MC: Mucinous carcinoma
ICC: Invasive cribriform carcinoma
IMPC: Invasive micropapillary carcinoma
CMF: Carcinoma with medullary features
